# The DNA cytosine deaminase APOBEC3H haplotype I likely contributes to breast and lung cancer mutagenesis

**DOI:** 10.1038/ncomms12918

**Published:** 2016-09-21

**Authors:** Gabriel J. Starrett, Elizabeth M. Luengas, Jennifer L. McCann, Diako Ebrahimi, Nuri A. Temiz, Robin P. Love, Yuqing Feng, Madison B. Adolph, Linda Chelico, Emily K. Law, Michael A. Carpenter, Reuben S Harris

**Affiliations:** 1 Department of Biochemistry, Molecular Biology and Biophysics, University of Minnesota, Minneapolis, Minnesota 55455, USA; 2 Masonic Cancer Center, University of Minnesota, Minneapolis, Minnesota 55455, USA; 3 Institute for Molecular Virology, University of Minnesota, Minneapolis, Minnesota 55455, USA; 4 Center for Genome Engineering, University of Minnesota, Minneapolis, Minnesota 55455, USA; 5 Department of Microbiology and Immunology, University of Saskatchewan, Saskatoon, Saskatchewan, Canada S7N 5E5; 6 Howard Hughes Medical Institute, University of Minnesota, Minneapolis, Minnesota 55455, USA

## Abstract

Cytosine mutations within TCA/T motifs are common in cancer. A likely cause is the DNA cytosine deaminase APOBEC3B (A3B). However, *A3B*-null breast tumours still have this mutational bias. Here we show that APOBEC3H haplotype I (A3H-I) provides a likely solution to this paradox. *A3B*-null tumours with this mutational bias have at least one copy of *A3H-I* despite little genetic linkage between these genes. Although deemed inactive previously, A3H-I has robust activity in biochemical and cellular assays, similar to A3H-II after compensation for lower protein expression levels. Gly105 in A3H-I (versus Arg105 in A3H-II) results in lower protein expression levels and increased nuclear localization, providing a mechanism for accessing genomic DNA. A3H-I also associates with clonal TCA/T-biased mutations in lung adenocarcinoma suggesting this enzyme makes broader contributions to cancer mutagenesis. These studies combine to suggest that A3B and A3H-I, together, explain the bulk of ‘APOBEC signature' mutations in cancer.

## Abstract (web summary)

The APOBEC family of enzymes are cytidine deaminases with APOBEC3A and APOBEC3B thought to contribute to DNA damage signatures detected in cancer genomes. Here, the authors demonstrate an unappreciated role for APOBEC3H haplotype I in the generation of DNA damage in breast cancer.

## Graphical abstract

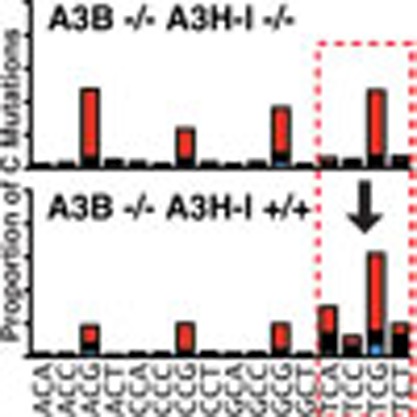

Tumour genome sequencing studies have identified a number of mutation patterns, or signatures, in cancer that directly reflect the source of the original DNA damage (reviewed by refs 1, 2, 3, 4). Prominent established sources include ultraviolet-induced C-to-T transition mutations in dipyrimidine motifs in melanoma (ultraviolet signature), and spontaneous, water-mediated methyl-C-to-T transition mutations in CG motifs in many tumour types (ageing signature), as well as a number of other known signatures. However, the most abundant previously unknown mutation signature to emerge from sequencing tumours is ‘APOBEC'. The signature of APOBEC mutagenesis is C-to-T transition and C-to-G transversion mutations within TCA and TCT trinucleotide motifs (hereafter TCW; TCG motifs may also be targeted by this process but are difficult to distinguish from the ageing signature and therefore discounted in most analyses). APOBEC signature mutations are mostly dispersed throughout the genome, but a fraction occurs in clusters called kataegis^5^. APOBEC signature mutations are prevalent in over half of human cancers and often account for the majority of mutations within a single tumour^5^,^6^,^7^,^8^,^9^,^10^,^11^,^12^. For instance, the APOBEC signature often dominates the overall mutation landscapes of breast, lung, head/neck, bladder and cervical cancers.

Although commonly referred to as a single entity, APOBEC is in fact a family of 11 distinct proteins in humans, and 9 have demonstrated DNA cytosine deaminase activity in a variety of assays with seven eliciting intrinsic preferences for TC motifs in single-stranded DNA (ssDNA; reviewed by refs 13, 14, 15). Leading candidates to account for the overall APOBEC mutation signature in cancer are APOBEC3A (A3A) and APOBEC3B (A3B). A3A has been attractive because it is arguably the most potent human DNA deaminase that, upon overexpression in heterologous systems, causes genomic DNA damage that results in cell death^7^,^16^,^17^,^18^,^19^,^20^,^21^. A comparison of the A3A and A3B mutation signatures derived from yeast overexpression experiments and the actual APOBEC signature from tumour genomes suggested that A3A provides the most parsimonious explanation^20^. However, endogenous A3A expression is largely specific to myeloid lineage cell types and, upon natural induction by interferon-α, it localizes to the cytoplasmic compartment and is not cytotoxic^22^,^23^,^24^,^25^. Consistent with strict developmental regulation and dedicated innate immune function, A3A has yet to be detected at the protein level in cancer cell lines or tumours. Moreover, much of the reported RNA level expression may be due to *A3B* sequence mismapping due to >90% nucleotide identity with *A3A*, a likelihood strongly supported by positive linear correlations between *A3A* and *A3B* expression in non-myeloid cancer data sets (Supplementary Fig. 1) and clear differences in the transcriptional regulatory programs for each of these genes^22^,^23^,^24^,^25^,^26^,^27^,^28^.

A3B was first implicated as an endogenous mutagen in breast cancer^7^. A3B is overexpressed in ∼50% of breast tumours and the majority of breast cancer cell lines^7^. A3B is constitutively nuclear^29^,^30^,^31^ and the only detectable DNA deaminase activity in breast cancer cell extracts^7^. A3B overexpression also induces DNA damage and cytotoxicity, but timing is delayed in comparison to A3A overexpression and more cells become multinucleated before dying^7^. *A3B* expression levels correlate positively with overall cytosine mutation loads in breast cancer^7^. *A3B* overexpression has also been documented in many other tumour types, although overexpression alone is not the sole factor determining whether or not a tumour will have a visible APOBEC mutation signature^7^,^8^,^9^,^10^,^32^,^33^. High *A3B* expression levels have been associated with poor clinical outcomes for oestrogen receptor-positive breast cancer, lung cancer, multiple myeloma and renal cell carcinoma^34^,^35^,^36^,^37^,^38^,^39^,^40^. Altogether, these data are consistent with a model in which A3B causes mutations that fuel the evolution of multiple different tumour types and negatively influences clinical outcomes^41^,^42^,^43^.

However, the importance of A3B in cancer has been questioned with the observation that APOBEC signature mutations are still clearly evident in *A3B*-null breast tumours^44^. A 29.5-kbp deletion that removes the entire *A3B* coding sequence and fuses the 3′ untranslated regions of *A3A* and *A3B* occurs at different frequencies in different populations around the world ranging from <5% in Caucasian and African populations to >30% in many Southeast Asian and Polynesian populations^45^. Here, we extend this result to the largest available breast tumour data sets, and test the hypothesis that stably expressed variants of the only other functionally dimorphic DNA deaminase family member, APOBEC3H (A3H), are responsible for APOBEC signature mutations in the absence of A3B.

A3H is the most polymorphic APOBEC3 protein in humans with seven reported haplotypes^46^,^47^. Haplotypes II, V and VII are stable, highly active enzymes with potent retrovirus restriction and hypermutation activities^46^,^47^,^48^,^49^,^50^. Haplotypes III, IV and VI are unstable proteins with no reported function, most likely due to a structure-corrupting deletion of Asn15 in the conserved α1-helix^46^,^47^,^48^,^49^. Haplotype I is poorly expressed at the protein level due to Gly105 (versus Arg105 in stable haplotypes) and, until now, has failed to show reproducible activity^46^,^47^,^48^,^49^,^51^. This functional dimorphism is apparent in immunoblots comparing overexpressed A3H constructs in 293 cells^47^ or endogenous A3H proteins in primary T lymphocytes^48^, in which the stable haplotypes show robust expression and unstable haplotypes have no detectable expression. An exception to these phenotypes is the A3H-I protein, which is expressed at steady-state levels ∼10-fold lower than stable variants and is difficult to detect by immunoblotting.

Surprisingly, during the course of testing the hypothesis that stable A3H haplotypes contribute to cancer mutagenesis, we found that the poorly expressed haplotype, A3H-I, associates with the APOBEC mutation signature in breast tumours lacking A3B. Linkage studies showed that the responsible SNP encoding Gly105, as well as surrounding genetic variations in *A3H*, are genetically unlinked to most of the remaining *APOBEC3* locus including *A3A* and *A3B*, indicating that other *APOBEC3* genes are unlikely to be responsible. Enzyme activity assays and HIV-1 restriction and mutation experiments demonstrated that A3H-I has strong DNA cytosine deaminase activity with a clear preference for TC dinucleotide substrates. Moreover, after compensation for lower protein expression levels, A3H-I and A3H-II showed similar enzymatic activities and local motif preferences. Subcellular localization images demonstrated that A3H-I has a significantly greater tendency to localize to the nuclear compartment in comparison to A3H-II, suggesting that Gly105 may disrupt an interaction with a cytoplasmic retaining factor and thereby provide a plausible mechanism for somatic mutation in cancer. The potential generality of this mechanism was indicated by an additional, statistically significant, correlation between A3H-I and early arising, clonal mutations in lung adenocarcinoma. Overall, together with prior studies on A3B, we propose that the combination of A3B and A3H-I explains the full APOBEC signature in breast and lung cancer, with a strong likelihood of extending more broadly to somatic mutation in other tumour types given the ∼50% global frequency of A3H-I, the general nature of the underlying molecular mechanism, and the fact that global searches have failed to identify an APOBEC-hypermutated tumour data set without at least one copy of either A3B or A3H-I.

## Results

### A3H-I associates with APOBEC signature mutations

**Figure 1:**
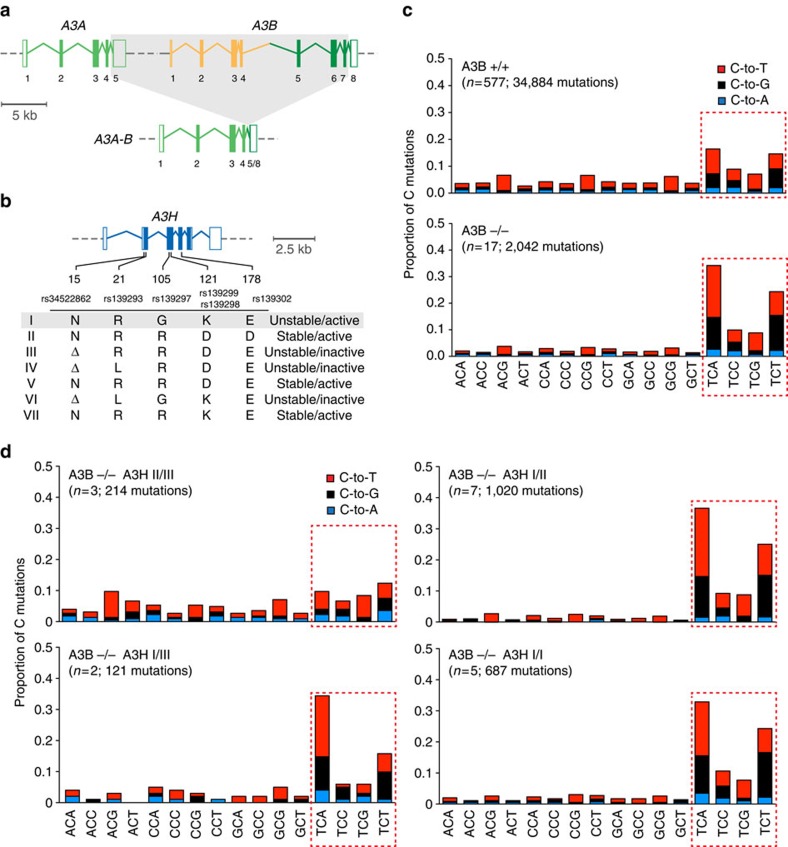
A3H haplotype I accounts for APOBEC signature mutations in *A3B*-null breast tumours. (**a**) Schematic of *A3A*, *A3B* and the *A3A-B* fusion gene. Exons are numbered and indicated by boxes, and coding regions are shaded. A 5 kbp scale is indicated. (**b**) Schematic of the *A3H* gene with haplotype-defining amino acid variants and SNP numbers listed below. Labelled as in **a**, except the scale indicates 2.5 kbp. (**c**) Bar plots depicting the proportions of cytosine mutations occurring in the indicated trinucleotide motifs in A3B^+/+^ and A3B^−/−^ breast tumors with *n*-values and total mutation numbers in parentheses. C-to-T, C-to-G and C-to-A are represented by red, black and blue shading, respectively. (**d**) Bar plots depicting the proportions of cytosine mutations occurring in the indicated trinucleotide motifs in A3B^−/−^ breast cancers with the indicated *A3H* haplotype combinations (*n*-values and total mutation numbers in parentheses). C-to-T, C-to-G and C-to-A are represented by red, black and blue shading, respectively.

To test the hypothesis that stable forms of A3H (haplotypes II, V and VII) contribute to breast cancer mutagenesis in the absence of A3B, we computationally screened and manually confirmed all available breast tumour data sets from TCGA to identify specimens with two copies of the *A3B* deletion allele, deduce *A3H* haplotypes and assess association with APOBEC signature mutations (gene schematics in Fig. 1a,b). A total of 17 *A3B*-null tumours were identified and, as reported previously from analyses of 13 null-tumours^44^, the overall APOBEC signature in these tumours is visibly more pronounced than the composite cytosine mutation spectrum from 577 breast tumours with two confirmed intact copies of *A3B* (boxed in Fig. 1c).

The 17 independent A3B-null tumours could be divided into 4 distinct A3H haplotype groupings for additional analyses. Contrary to our initial hypothesis, *A3B*-null tumours with an A3H-II/III haplotype combination, one active allele and one inactive allele, showed the lowest (not the highest) proportion of TC-biased cytosine mutations (Fig. 1d). This result indicated that A3H-II is unlikely to be contributing to the APOBEC mutation signature in breast cancer. We were additionally surprised to find that *A3B*-null tumours with one copy of A3H-I (A3H-I/II or I/III) or two copies of A3H-I showed remarkably strong APOBEC signatures (boxed in Fig. 1d with statistical significance for non-A3H-I versus A3H-I mutation proportion comparisons in Supplementary Table 1). This relationship was especially strong and statistically significant for C-to-T transitions and C-to-G transversion mutations in TCA trinucleotide contexts, which represent the largest proportion of APOBEC signature mutations in cancer. These results suggested that the previously classified ‘unstable' and ‘inactive' A3H-I protein could be the source of APOBEC signature mutations in breast tumours lacking A3B.

### *A3H* linkage analysis

We next performed a genetic linkage analysis using all available 1000 genomes SNP data within the 7-gene human *APOBEC3* locus, arranged tandemly *A3A* through *A3H* in ∼120 kbp segment of chromosome 22, to assess the simple possibility that the *A3B* deletion and *A3H-I* allele may be linked genetically and, therefore, that one of the other family members in the same linkage group may be responsible for the observed APOBEC signature mutations in *A3B*-null, *A3H-I* breast tumours. SNP data from the 1000 genomes project^52^ were used to determine linkage disequilibrium (*r*^2^) between all *A3H* SNPs and all SNPs for the six other *APOBEC3* genes. The *r*^2^ values for linkage disequilibrium between two SNPs range from 0 for no correlation to 1 for a perfect correlation. An *r*^2^ of 0.9 indicates that knowledge of one allele will correctly predict the second allele 90% of the time.

**Figure 2:**
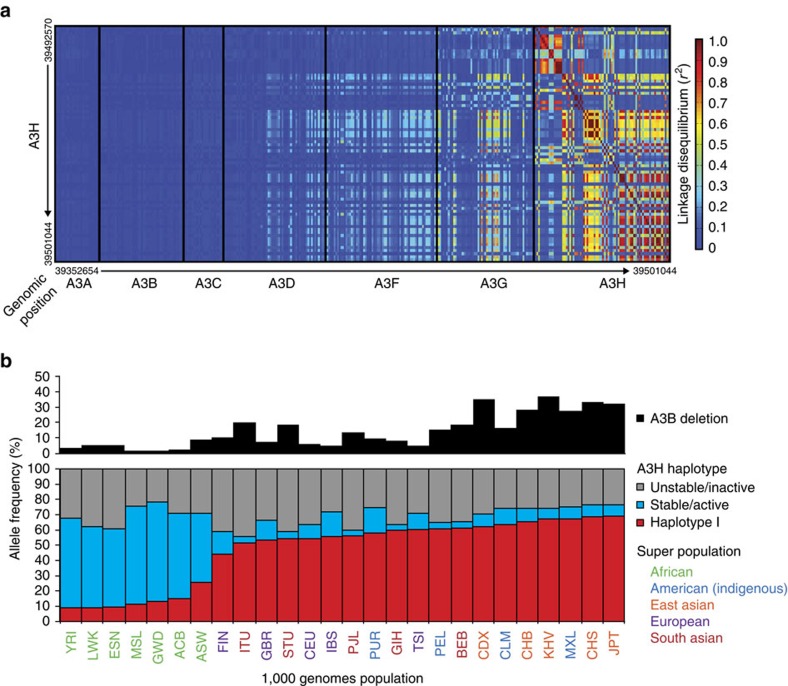
Polymorphisms in *A3H* are not in linkage disequilibrium with *A3A* or *A3B*. (**a**) Heatmap showing the strength of linkage (*r*^2^) of SNPs located within the *A3H* gene versus the rest of the *APOBEC3* locus. (**b**) Bar plots of the *A3B* deletion and *A3H* haplotype frequencies for the indicated populations (A3H-I in red; stable A3H-II/V/VII in blue and unstable A3H-III/IV/VI in grey). Superpopulations are colour coded for visualization of larger geographic areas, and individual 3-letter population identifiers are from the 1000 genomes project.

Interestingly, we found that the *A3H* gene is only linked strongly to itself and to the immediate upstream gene, *A3G*. Linkage is weaker with genes further upstream, *A3D* and A3F, and completely absent with *A3A*, *A3B* and *A3C* (Fig. 2a). Although *A3G* is linked to *A3H*, it is very unlikely to be involved in cancer mutagenesis because the encoded enzyme has an intrinsic preference for the 3′ cytosine in CC and CCC motifs^53^,^54^, which are rarely mutated in cancer (for example, Fig. 1 for cytosine mutations in breast cancer). Importantly, this linkage analysis shows that the *A3H* gene is unlinked to *A3A* (or the *A3A*-*B* chimeric gene resulting from the *A3B* deletion), suggesting that the significant association with *A3H-I* and APOBEC signature mutations in *A3B*-null tumours may be due directly to A3H-I enzymatic activity and not to A3A (as favoured by recent studies^7^,^16^,^17^,^18^,^19^,^20^,^21^) nor to any other TC-preferring enzyme encoded by the locus.

Our analyses of the 1000 genomes project data also revealed an interesting inverse correlation between the presence of *A3B* and *A3H-I* in different human populations around the world (−0.76, Spearman's rho; *P*=0.0015; Fig. 2b). In areas such as Africa where *A3B* dominates (>95%), *A3H-I* occurs at a very low frequency (<10%). In other parts of the world, such as Southeast Asia where the *A3B* deletion allele is found at higher frequencies (>30%), *A3H-I* is found at higher frequencies (∼65%). In other words, if *A3B* is present then *A3H-I* is rare, and if *A3B* is absent then *A3H-I* is more common. This correlation implies that *A3B* and *A3H-I* may have a redundant physiological function, possibly in antiviral immunity, or that the two genes together may incur a fitness penalty. In addition, when *A3H-I* is common then *A3H-II* is rare (Southeast Asia), whereas when *A3H-I* is rare then *A3H-II* is common (Africa). The null *A3H* haplotypes (III/IV/VI) occur at similar frequencies regardless of population implying the existence of an as yet unknown balancing selective pressure.

### A3H-I is an active DNA cytosine deaminase

Prior studies comparing the DNA deamination activity of A3H-I and A3H-II have reported that the former protein is inactive^46^,^47^,^51^ or weakly active^48^. Obviously, an inactive enzyme cannot contribute to cancer mutagenesis. We therefore interrogated the activity of this enzyme using multiple independent approaches.

**Figure 3:**
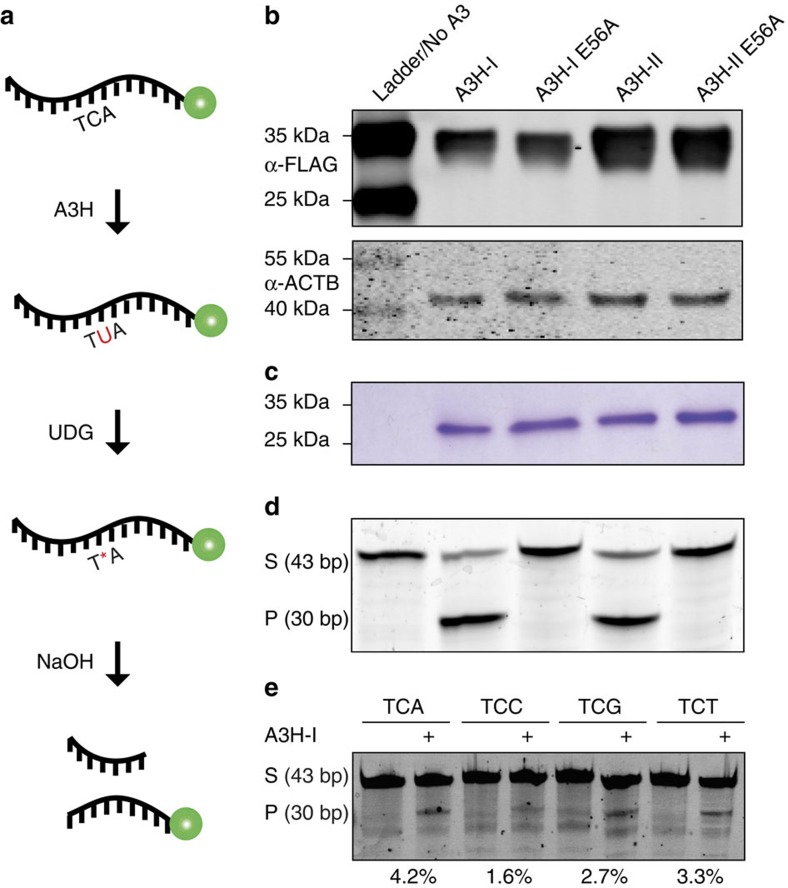
A3H haplotype I is an active DNA cytosine deaminase. (**a**) Schematic of the ssDNA deamination assay. A3H-mediated deamination yields a uracil that, on excision by excess uracil DNA glycosylase, is converted into a hydroxide-labile abasic site. (**b**) Anti-FLAG immunoblot of A3H-I, A3H-II and catalytic mutant derivatives expressed in 293T cells prior to purification, with an anti-ACTB immunoblot shown below as a loading control. (**c**) Image of a Coomassie-stained gel with approximately equal amounts of A3H-I, A3H-II and catalytic mutant derivatives purified from 293T cells. (**d**) Activity data for the recombinant A3H proteins shown in **c** (P, product; S, substrate). (**e**) Activity of GST-A3H-I purified from insect cells using the indicated trinucleotide containing ssDNA substrates (P, product; S, substrate).

A3H-I, A3H-II and catalytic mutant derivatives (E56A) were purified from human 293T cells, normalized to be equimolar and assayed for DNA cytosine deaminase activity using a gel-based ssDNA deamination assay^7^,^27^,^32^,^55^,^56^ (schematic in Fig. 3a). In agreement with prior studies^46^,^47^,^48^,^49^,^51^, A3H-I showed lower overall protein expression levels in cellular lysates in comparison to A3H-II (Fig. 3b). However, similar concentrations of each protein could be achieved by concentrating A3H-I, as evidenced by near-equivalent A3H band intensities on direct visualization of SDS–polyacrylamide gel electrophoresis fractionated proteins (Fig. 3c). A head-to-head comparison of A3H-I and A3H-II using a ssDNA with a single TCA target motif indicated near-equivalent enzymatic activities (Fig. 3d). Importantly, the E56A catalytic mutant derivatives purified and analysed in parallel had no detectable catalytic activity, which demonstrates that all of the observed activity is due to active A3H-I or A3H-II (and not, for instance, to a co-purifying factor from 293T cells). In independent experiments, recombinant A3H-I from *Sf*9 insect cells also elicited ssDNA C-to-U editing activity indicating no other human factors are required (Fig. 3e).

**Figure 4:**
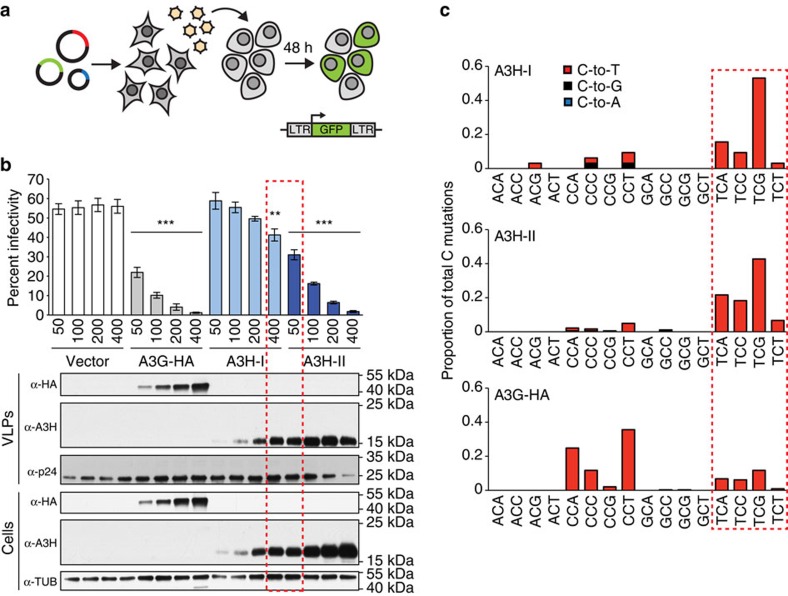
A3H haplotype I has enzymatic activity against viral DNA. (**a**) HIV-1 infectivity assay. Viral and A3 expression vectors are transfected into 293 producer cells and, after 48 h, virus-containing supernatants are titered by infecting CEM-GFP reporter cells in which an integrated LTR-GFP cassette is activated by the Tat protein expressed from newly integrated viruses. Infectivity is quantified by flow cytometry and calculating the percentage of GFP-positive reporter cells. (**b**) Mean and s.e.m. plotted for three biological replicates of HIV-1 infectivity data for Vif-deficient viruses produced in 293 cells expressing a vector control, A3G-HA, A3H-I or A3H-II (*P<0.05, **P<0.01, ***P<0.001, Welch's two sided *t*-test). Immunoblots for the indicated proteins in cell lysates and virus containing supernatants are shown below. (**c**) C-to-T mutation distribution in viral DNA sequences recovered from CEM-GFP reporter cells. C-to-T, C-to-G and C-to-A are represented by red, black and blue shading, respectively. The mutations are reported for the viral cDNA strand, rather than the conventional genomic strand to facilitate comparisons with tumours.

Next, HIV-1 restriction and mutation assays were used as biological read-outs for A3H-I activity (schematic in Fig. 4a). Vif-deficient HIV-1 particles were produced in 293T cells with a range of untagged A3H concentrations up to maximally tolerated amounts. As expected^13^,^14^, A3H-II and A3G-HA caused strong dose-responsive decreases in virus infectivity (Fig. 4b). In comparison, overexpression of A3H-I caused more modest, but still significant, drops in virus infectivity (200 ng, *P*=0.055; 400 ng, *P*=0.0059, Welch's *t*-test). These virus restriction phenotypes correlated with the overall amounts of A3H and A3G-HA proteins expressed in cells and packaged into nascent viral particles (Fig. 4b). It is notable that the highest quantity of A3H-I yielded an HIV-1 restriction phenotype similar to the lowest amount of A3H-II, and that these transfected amounts produced similar steady-state protein levels by immunoblotting (red boxed data in Fig. 4b). These HIV-1 restriction phenotypes lend further support to the *in vitro* biochemical results above and to the surprising finding that A3H-I elicits a level of catalytic activity similar to the better-expressed A3H-II enzyme.

To extend these results, DNA was purified from infected target cells (400 ng condition), and high-fidelity PCR was used to amplify proviral sequences for mutation analyses. A3H-I and A3H-II inflicted an average of 3.4 and 18 C-to-T mutations per kilobase, respectively (>32 independent 276 bp sequences per condition). Interestingly, although A3H-I has lower virus restriction activity per unit of transfected expression plasmid, its intrinsic DNA cytosine deamination preferences strongly resemble those of A3H-II with clear biases for TC dinucleotides (Fig. 4c). As expected, A3G caused high levels of mutation within CC dinucleotide contexts, which are rarely mutated in cancer. It is noteworthy that the APOBEC3-catalysed viral cDNA uracils in this system are not subject to normal cellular DNA repair processes because DNA deamination and reverse transcription occur within the physical confines of the capsid-encased viral core, and reverse transcription effectively immortalizes these uracil lesions as viral genomic strand G-to-A mutations before integration into the genomic DNA of a susceptible host cell (mechanism reviewed by refs 13, 14, 15).

### A3H-I has increased nuclear localization

We next asked if A3H-I is capable of accessing the nuclear compartment, which is another property likely to be essential for cancer mutagenesis. Prior studies have lacked consensus with epitope-tagged A3H-I showing variable subcellular localization^46^,^49^,^51^,^57^. However, one study proposed that A3H-II (Arg105) is retained in the cytoplasm by interacting with a specific host factor, and that the Gly105 amino acid characteristic of A3H-I dislodges this mechanism and enables entry into the nuclear compartment by passive diffusion^49^.

**Figure 5:**
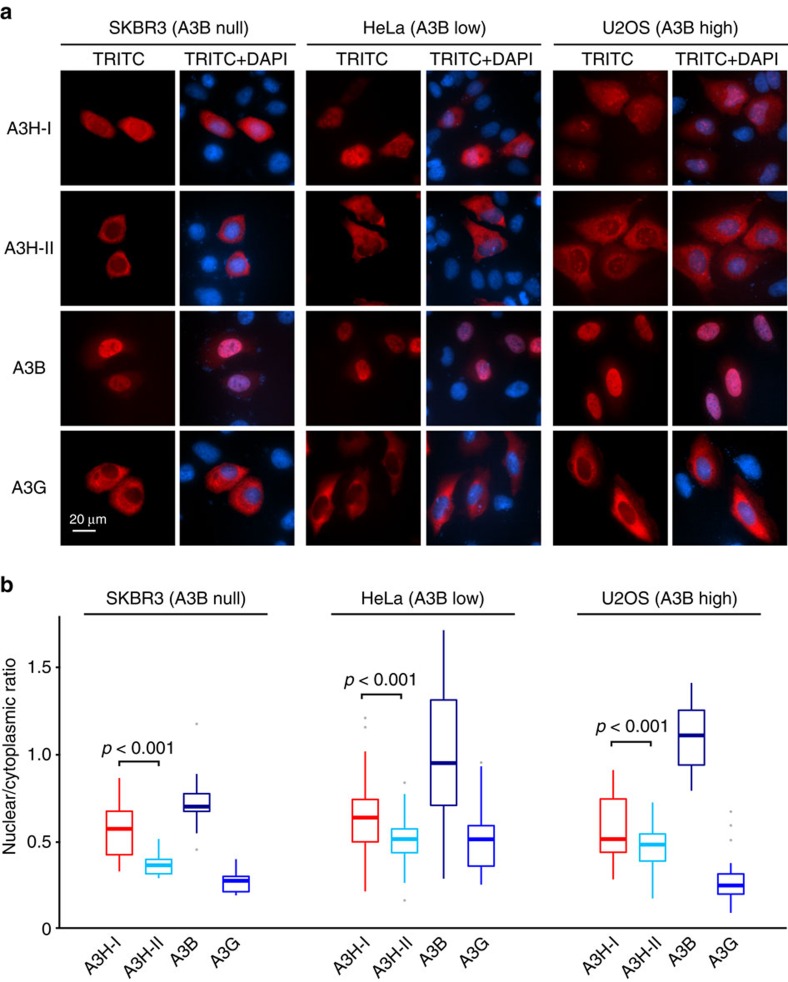
A3H haplotype I has greater nuclear localization than haplotype II. (**a**) Representative images of A3H-I (untagged), A3H-II (untagged), A3B-HA and A3G-HA in SK-BR-3, HeLa and U2OS cells. The 20 μm scale applies to all images. (**b**) Whisker plots quantifying the subcellular localization data as nuclear-to-cytoplasmic ratios for *n*>50 cells per condition. The average is shown, the error box represents the first and third quartiles, and the whiskers extend to the highest value within 1.5 × the interquartile range (*P* values determined by two-tailed Welch's *t*-test).

To clarify and advance this important point, we used immunofluorescent microscopy to quantify the localization of untagged A3H-I versus A3H-II in cell lines with varying endogenous A3B levels (null, SK-BR-3; low, HeLa; high, U2OS)^7^. A3B-HA and A3G-HA were used as controls for predominantly nuclear and cytoplasmic localization, respectively (Fig. 5). We found the overall subcellular distributions of A3H-I and A3H-II to be consistent in all cell types, regardless of endogenous A3B levels, with the former enzyme invariably appearing more nuclear than the latter (representative images, Fig. 5a; quantification, Fig. 5b). These data show that A3H-I is proficient at entering the nuclear compartment, and advance the general model in which Gly105 disrupts a cytoplasmic retention mechanism and enables the A3H-I enzyme to breach the nuclear compartment and mutagenize genomic DNA.

### A3H-I explains many clonal mutations in lung adenocarcinoma

**Figure 6:**
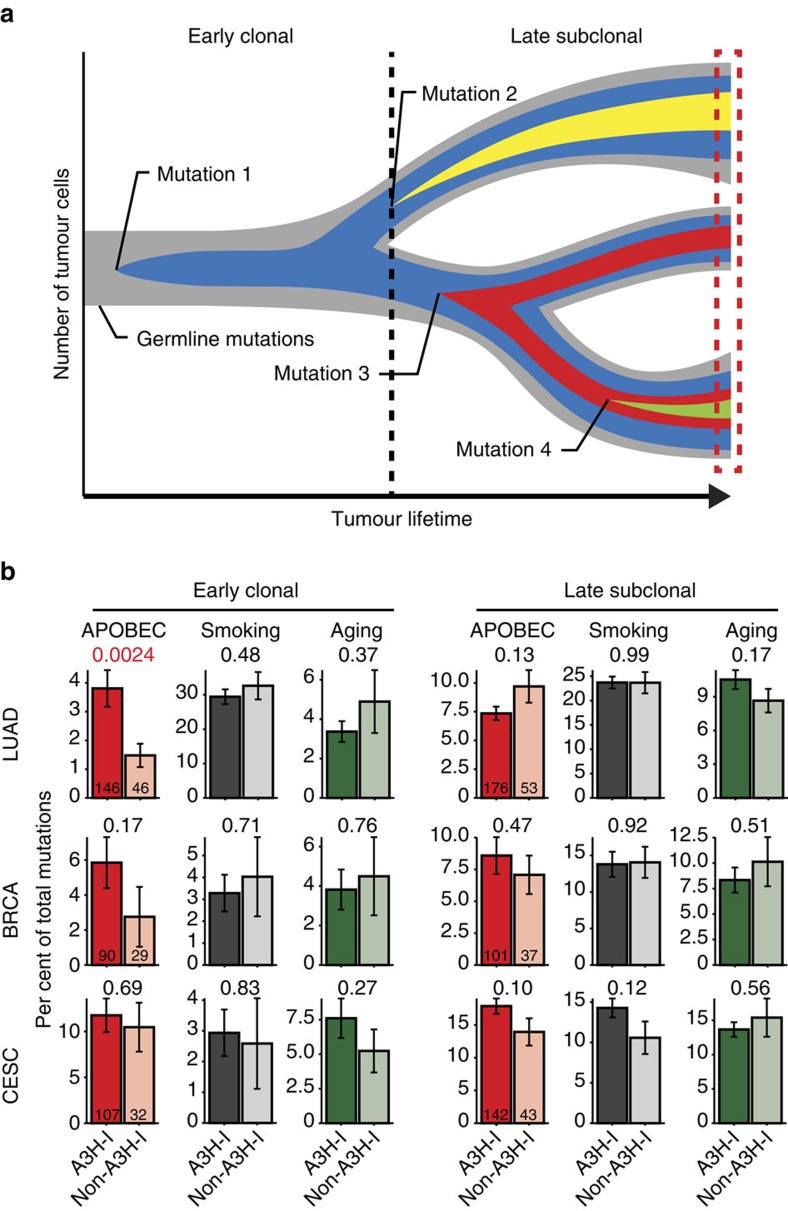
A3H haplotype I contributes APOBEC signature mutations to lung cancer. (**a**) Tree-based cartoon of tumour evolution. To represent the heterogeneity of each tumour deep sequencing data set (boxed area), 1 trunk mutation (blue) and 3 branch mutations (other colours) are depicted on a background of normal germline DNA (grey). Trunk mutations occur early and are found in every tumour branch, whereas branch mutations occur later in one or more branches (that is, clonal versus subclonal). (**b**) Bar plots showing the frequency of clonal and subclonal mutations in the indicated cancer types attributable to APOBEC, smoking and ageing (BRCA, breast cancer; CESC, cervical cancer; LUAD, lung adenocarcinoma). Each bar represents the average proportion ±SEM of signature mutations occurring within each *A3H* haplotype group (that is, *A3H-I* versus non-*A3H-I*). The total number of tumours with 1 or 2 copies of G105 (A3H-I) or 2 copies of R105 (A3H-II and other haplotypes) is indicated within the first set of histogram bars. Welch's two-tailed *t*-test for each category was used to calculate the *P* value above each graph.

Given the results detailed above, particularly the potent enzymatic activity of A3H-I with an intrinsic preference for cytosines in a TC context, the capacity of A3H-I to mutate a variety of substrates, and the ability of A3H-I to breach the nuclear compartment, we next performed a comprehensive analysis of all available TCGA tumour data sets with *n*-values >400 exomes to begin to assess whether A3H-I is a general source of mutation in cancer or a unique mechanism that compensates for the loss of A3B in a limited subset of breast cancers. To distinguish between these possibilities, we determined whether A3H-I associates with common mutation signatures including APOBEC, smoking and ageing (respectively, C-to-T in TCW motifs, C-to-A in any motif and C-to-T in CG motifs). We predicted an association of A3H-I with APOBEC signature in some tumour types, but not with smoking or ageing in any tumour type because these signatures are clearly due to independent mechanisms. Mutations with the highest frequency of occurrence in a given tumour normalized by copy number and tumour purity are considered early arising and clonal, whereas any mutation occurring at a lower frequency is considered later-arising and subclonal (Fig. 6a). Such temporal relationships are critical because different mutational processes have been shown to promote different stages of tumour evolution^3^.

Three distinct scenarios emerged from these analyses (Fig. 6b and Supplementary Fig. 2). In the first, A3H-I associates with the occurrence of APOBEC signature mutations despite contributions from multiple mutational processes. Nearly threefold more early-clonal APOBEC-signature mutations were evident in lung adenocarcinomas with at least 1 copy of A3H-I in comparison to tumours with any other A3H haplotype (*P*=0.0024, Welch's two-tailed *t*-test; top left histogram in Fig. 6b). In contrast and as expected, clonal mutation signatures attributable to smoking and ageing did not correlate with A3H haplotype (*P*=0.48 and *P*=0.37, respectively). Subclonal APOBEC-signature mutations also occurred independently of A3H haplotype (*P*=0.13) and, based on prior studies^6^,^8^,^9^,^10^, are most likely due to the temporally late upregulation of A3B. Clonal APOBEC signature mutations also appeared to be enriched in breast tumours with A3H-I versus those with any other haplotype, but this difference is not statistically significant (*P*=0.17, Welch's two-tailed *t*-test). This result could easily be due to A3B upregulation occurring at variable times during the progression of individual breast tumours because the trend completely disappears in the analysis of subclonal APOBEC signature mutations where A3B has already been implicated strongly^5^,^6^,^7^,^8^,^9^,^10^,^11^,^12^.

In the second scenario, both clonal and subclonal APOBEC mutation signatures are stronger, as judged by the higher percentages of total cytosine mutations, and there are no correlations with the presence or absence of A3H-I. The clearest example is cervical cancer, where human papilloma virus (HPV) infection is an established early event^58^, and HPV infection has been mechanistically linked to A3B upregulation^27^,^33^. Here, ∼10% of overall APOBEC signature mutations are clonal for both A3H-I and non-A3H-I cervical tumours, and this proportion rises to nearly 15% for APOBEC signature subclonal mutations (*P*=0.69 and *P*=0.10, respectively, Welch's two-tailed t-test; Fig. 6b). Thus, in this virus-induced scenario, we propose that chronic levels of A3B eclipse any mutational contributions from A3H-I or, alternatively, that the *A3H* gene is not expressed at the messenger RNA level in these tumour types (supported by cell-based HPV studies^27^). Again, as expected, both clonal and subclonal smoking and ageing mutation signatures are unrelated to A3H haplotype.

In the final scenario, a proportion of cancer types simply do not manifest an APOBEC mutation signature^6^,^8^,^9^,^11^,^12^,^59^. For instance, prostate cancer and low-grade glioma lack an APOBEC signature at any time point (Supplementary Fig. 2). There are many possible molecular explanations for why the general mutation mechanism described here for A3H-I may not be operational in these cancer types. The simplest is that both basal and induced levels of *A3H-I* messenger RNA expression are likely to be constrained by cell type and developmental programme, and therefore differences between protein-level haplotypes may be irrelevant and incapable of contributing to the overall mutation spectrum.

## Discussion

The studies presented here are the first to implicate A3H-I as an enzymatic contributor to ‘APOBEC signature' mutations in breast and lung cancer, with a general mechanism that could extend to other cancer types. This work was initiated with the goal of providing a molecular explanation to the paradox that APOBEC signature mutations still exist in *A3B*-null breast cancers^44^. We had independently reached and expanded on the same conclusion (data summarized in Fig. 1c), and we set out to test the hypothesis that stably expressed variants of the only other functionally dimorphic APOBEC3 family member, A3H, would be responsible. To our surprise, stable *A3H* haplotypes did not explain the APOBEC signature mutations in *A3B*-null tumours; instead, a variant previously deemed unstable and inactive, *A3H-I*, showed a statistically significant association (Fig. 1d). A linkage analysis further strengthened this possibility and, simultaneously, discounted participation by other APOBEC3 family members including wild-type A3A and A3A produced by the chimeric *A3A-B* fusion gene generated by the 29.5 kbp *A3B* deletion (Fig. 2). Biochemical and cell-based studies contrasted with prior reports and clearly demonstrated that A3H-I is not only catalytically active but, once protein concentrations were equalized, it also proved similarly active to the A3H-II enzyme and yielded a near-identical TC-biased ssDNA deamination preference (Figs 3, 4). Subcellular localization studies revealed the likely molecular mechanism responsible for enzymatically active A3H-I gaining access to the nuclear compartment, with A3H-I Gly105 disrupting a likely cytoplasmic retention mechanism (Fig. 5). Finally, an informatics approach was used to significantly associate A3H-I with clonal APOBEC signature mutations in lung cancer (Fig. 6). The high frequency of A3H-I in global populations, 48% (54% in Caucasians) based on 1000 genome project data^52^, and the general nature of the mechanism described here (mislocalization of an active DNA cytosine deaminase) combine to suggest relevance to other cancer types.

As described in the Introduction section, previous work has favoured A3A and/or A3B as the predominant sources of the APOBEC signature mutation in cancer. The studies presented here are the first to implicate A3H-I and, simultaneously, further support A3B and cast doubt on a possible role of A3A and other APOBEC family members in cancer mutagenesis. For instance, if A3A or another family member caused the APOBEC signature in *A3B*-null tumours, then statistically significant associations with *A3H-I* would never have emerged from the comprehensive analyses in Fig. 1 for breast cancer and Fig. 6 for lung cancer. Moreover, because the APOBEC signature is not found in *A3B*-null/non-*A3H-I* breast tumours (Fig. 1d), it is possible that A3B and A3H-I account for all APOBEC signature mutations in breast cancer. In support of this idea, an exhaustive analysis of all TCGA tumour data sets failed to find a single tumour with an APOBEC mutation signature that did not have either one copy of A3B or A3H-I (*n*=6,863 tumours over 15 cancer types). Thus our studies are also the first to positively link A3B and A3H-I at the genetic level to the APOBEC mutation signature observed broadly in cancer. Future population-focused studies will either confirm these data or unambiguously implicate another APOBEC enzyme (for example, analyses of A3B-null/A3H-inactive (haplotypes III, IV and VI) tumours could be informative although identifying cohorts with statistically significant numbers will be non-trivial).

Many factors contribute to the composite mutation spectra observed in cancer and comparisons with models systems are, at worst, misleading and, at best, challenging and inaccurate. For instance, the APOBEC signature of cytosine mutations in TCA and TCT motifs (Fig. 1) is the net result of the intrinsic ssDNA preference of the deaminase^55^,^60^,^61^ and the biased uracil excision activity of nuclear UNG2 (likely also influenced by contributions from other uracil DNA glycosylases)^62^,^63^, and the biased insertion of adenine or cytosine bases opposite abasic sites by DNA polymerization enzymes (most DNA polymerases follow the A-rule with the exception of REV1, which preferentially inserts cytosine and accounts for APOBEC-induced C-to-G transversions)^64^,^65^,^66^. The biochemical DNA deamination preferences with recombinant A3H-I in Fig. 3d are concordant with the observed APOBEC signature mutations in cancer with TCA/T/G targets preferred over TCC. Although A3H-I (this study) and A3B (refs 7, 32, 55) can efficiently deaminate cytosines in TCG, mutations in tumours within this trinucleotide context are numerically less common than those in TCA/T because CpG motifs are underrepresented in exomic regions of the human genome and methylation of these motifs decreases the efficiency of enzymatic deamination *in vitro* by at least fivefold^56^,^67^,^68^ (further complicated by the fact that methylation increases the efficiency of spontaneous hydrolytic deamination^69^). However, if a correction factor for trinucleotide motif frequency is applied to the observed mutation frequencies in Fig. 1d, then cytosine mutations within TCG motifs become enriched relative to the abundance of this motif the human genome, fully consistent with the biochemical preferences of A3H (Supplementary Fig. 3). A3H-catalysed mutation in Vif-deficient HIV occurs most frequently within TCG and TCA motifs and least frequently within TCC and TCT motifs, and this result becomes even clearer on examination of weighted mutation frequencies (Supplementary Fig. 4) and more closely resembles the weighted mutation frequencies of the breast tumour data sets (Supplementary Fig. 3). These results do not exactly mirror the biochemical data, likely because many experimental variables differ (enzyme sources, substrates, reaction conditions and so on). Nevertheless, each of our different experimental series still contributed an important piece to the overall picture, and the biochemical approach was invaluable for demonstrating that A3H-I is active and has essentially the same intrinsic DNA deamination preferences as A3H-II.

How can prior work be further reconciled? One recent study compared mutation patterns induced by A3A and A3B in yeast with the actual APOBEC signature in human cancer and, relying heavily on comparing frequencies of the −2 base relative to the mutated cytosine, concluded that A3A was 10-times more important than A3B in cancer mutagenesis^20^. Unfortunately, this work did not consider A3H and other human APOBEC3 enzymes preferring TC substrates. As discussed above, it difficult to account for the fact that yeast and human genomic structures and DNA repair processes differ and that this is likely to strongly influence the distribution of initial uracil lesions that ultimately become mutations. Another recent paper also favoured A3A and advanced the idea that the *A3A-B* chimeric gene created by the 29.5 kbp *A3B* deletion disrupts *A3A* regulation and results in elevated A3A protein levels, genomic DNA damage and cancer mutagenesis^19^. If this were the case then A3H-I would not have shown statistically significant associations with breast and lung cancer APOBEC mutation signatures (Figs 1 and 6). This study also relied heavily on analysis of transfected reporter constructs (not endogenous *A3A*) and failed to do the critical cause/effect experiment of specifically depleting endogenous *A3A* expression in *A3B*-null SK-BR-3 breast cancer cells and asking if it alone is responsible for the DNA damage phenotypes caused by PMA treatment. We investigated this possibility using CRISPR to recreate the human *A3B* deletion in an isogenic system, the breast cancer cell line MCF-7, and found no significant change in expression of the *A3A-B* fusion gene after deleting *A3B* (Supplementary Fig. 5). An additional critical point is that many studies purporting to document *A3A* expression in tumours may be undermined by *A3B* RNAseq reads mismapping to *A3A* and/or by *A3B* cDNA cross-hybridizing to *A3A* probes on microarrays and/or by *A3A* expressing immune cells infiltrating the analysed tumour tissues^7^,^20^,^32^,^70^ (Supplementary Fig. 1). Accordingly, to our knowledge, no studies to-date have documented A3A protein or enzymatic activity in primary tumour samples or tumour-derived cell lines. However, despite mounting evidence against A3A and other family members, we acknowledge that it is currently difficult to eliminate the possibility that one or more of these proteins may be able to contribute to cancer mutagenesis (for example, in a tumour type not addressed here due genetic and statistical constraints of TCGA data sets).

**Figure 7:**
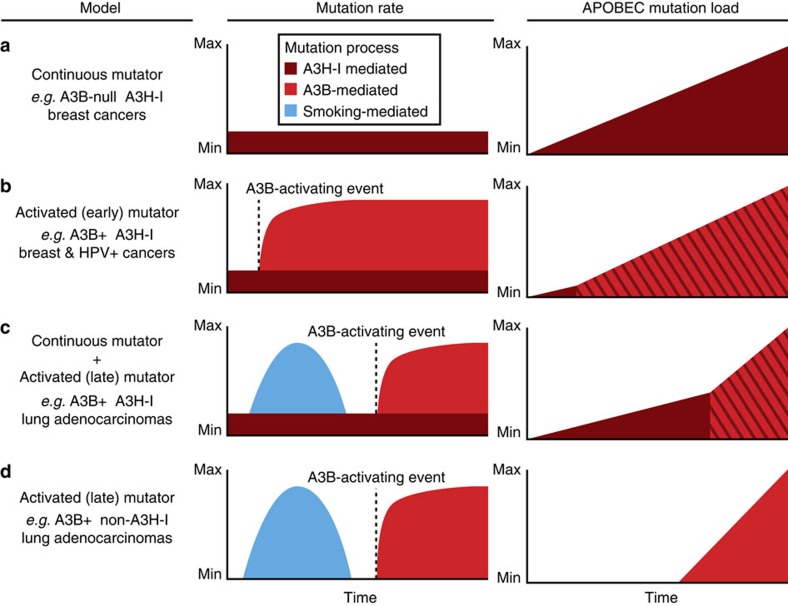
Models for differential APOBEC mutation accumulation in cancer. The far left column describes the *A3B* and *A3H-I* genotypes of each model as well as examples of relevant tumour types. The middle columns show the average mutation rate over time for each model with sources of mutations highlighted in different colours, smoking (blue), A3B (red) and A3H-I (maroon). The far right column depicts the accumulation of somatic APOBEC signature mutations over time, with mutations mediated by A3B and A3H-I represented in red and maroon, respectively. Somatic mutations from both APOBEC3 enzymes are shown as red/maroon diagonal stripes to highlight that these mutations are not easily distinguishable. (**a**) The continuous mutator model depicts constant A3H-I mediated mutagenesis and subsequent accumulation of APOBEC-signature mutations over time in the absence of A3B as may be occurring in some breast cancers. (**b**) The activated (early) mutator model depicts a rapid increase in A3B-mediated mutations and APOBEC signature mutations after an A3B-activating event such as HPV-infection in cervical cancers or a currently unknown mechanism in breast cancers. (**c**) The continuous mutator plus activated (late) mutator model depicts the constant accumulation of APOBEC-signature mutations mediated by A3H-I as shown in **a**. For contrast, the distinct contribution from smoking-mediated mutagenesis (blue) is shown as an early finite time period. Late activation of A3B then leads to a more rapid accumulation of APOBEC signature mutations over time effectively eclipsing the A3H-I contribution. (**d**) The activated (late) mutator model is nearly identical to the model shown in **c**, however the absence of A3H-I results in no early APOBEC-signature mutations as may be occurring in some lung adenocarcinomas.

Our results suggest distinct temporal models for the generation of APOBEC signature mutations in cancer. In the first, in *A3B*-null cancers such as in a subset of breast tumours described here, A3H-I may provide a low mutator activity that over a long period of time results in the observed APOBEC signature mutation spectrum and load (continuous mutator model in Fig. 7a). This mutation programme may be prone to periodic ‘flares' (not depicted) because at least one virus (HIV-1) has been shown to induce A3H expression in primary cells^48^,^71^, and other viruses may have similar stimulatory effects. This model may be particularly relevant to Southeast Asian populations with high frequencies of both *A3H-I* and the *A3B* deletion allele (Fig. 2). In the second model, in A3B overexpressing tumours such as many breast cancers and HPV-positive cancers, the mutational impact of A3B may be early, strong, constitutive and additive to that of A3H-I, and the powerful effect of A3B and its similar TC target preferences may rapidly eclipse the A3H-I contribution (activated (early) mutator model in Fig. 7b). HPV infection provides a mechanism for A3B upregulation in virus-positive tumour types, but the mechanisms responsible for early A3B induction in virus-negative tumours are less uniform and less clear (for example, ref. 26). In the third model, the impact of A3H-I is evident among early-arising clonal mutation spectrum in lung adenocarcinomas but it eventually becomes eclipsed by A3B overexpression at a later point in tumour development (continuous mutator plus activated (late) mutator model in Fig. 7c). In a variant of this model, the early continuous mutator effect is absent in tumours lacking A3H-I (activated (late) mutator model in Fig. 7d). In all of the models, apart from those depicting an early smoking signature, other prominent sources of mutation are excluded for purposes of focusing on the APOBEC signature and the different contributions of A3H-I and A3B observed in this study. Such additional sources of mutation are of course capable of contributing to the overall mutation loads and spectra in various tumour types. These models may extend to APOBEC signature cancers beyond those highlighted here, and future studies should be designed to isolate and quantify the mutagenic contributions of A3H-I, A3B and possibly other family members. Future studies should also examine the clinical impact of these different mutational sources together, as well as in isolation, in appropriate populations. Indeed, an analysis of lung cancer incidence in China indicated that unstable/inactive forms of A3H may be protective^72^ and, therefore, we suggest that A3H-I could be a significant risk factor as the predominant allele in China (∼70%) and other regions of the world.

## Methods

### *APOBEC* genotyping and tumour mutation analyses

TCGA RNAseq and WES data were obtained between April 2014 and January 2016 (https://cghub.ucsc.edu/). Somatic mutation and CNV data were obtained in January 2016 (https://confluence.broadinstitute.org/display/GDAC/Download). Initial candidate *A3B*-null patients were identified using TCGA SNP CNV data with a sample segment mean of -1.5 or less with probe start positions 37693565, 37693563 or 37693530 on chromosome 22. Additional *A3B*-null candidates were identified using WES of the *A3A* and *A3B* genes and flanking exons preserved with the deletion allele. *A3B*-null patients were confirmed by manual inspection of WES alignments (for example, Supplementary Fig. 6). A3H genotypes were determined by extracting base calls, quality and coverage data from WES alignments spanning the *APOBEC* locus for tumour/normal samples acquired from the TCGA. Global *A3H* allele frequencies were estimated using phased variants from all available data as of December 2015 from 1000 Genomes Project^52^.

All somatic mutations were isolated with adjacent 5′ and 3′ nucleotides using the hg19 reference genome. Mutations were binned by trinucleotide context and proportions calculated compared with total somatic mutations. No statistical difference in the total mutation loads were evident between the various genotypic groups of *A3B* and *A3H* breast tumours, which is not particularly surprising given the fact that the durations of tumour growth are unknown (Supplementary Fig. 7). To take motif abundance into consideration, we first determined the frequency of each trinucleotide motif in the reference sequence by counting each observed motif and dividing by the total number of trinucleotide motifs possible in the reference (length of sequence - 2). Counts of mutations at each trinucleotide motif were then divided by the previously calculated frequency of that trinucleotide in the reference and these adjusted counts were divided by the sum of all adjusted counts to derive weighted frequencies.

The chronological timing of somatic mutations was determined using published methods and definitions^10^,^73^ with conservative modifications. Specifically, tumour purity as calculated by ESTIMATE from TCGA RNAseqV2 and GISTIC2 copy-number calls from Broad were used instead of ASCAT data to adjust variant allele frequencies^74^. 95% confidence intervals (CI) of adjusted allele frequencies were determined by resampling the number of reads supporting the reference and alternate alleles at half of the observed coverage (10,000 bootstraps). Early-clonal mutations were any mutation occurring clonally before a copy number altering event. This was defined as a mutation with an adjusted allele frequency lower 95% CI that is >1 copy (that is, AF>0.50) in diploid regions of the genome and any mutation that corresponds to 2 or more copies in regions of copy number amplifications (for example, CN=3, AF=0.667). Late-clonal mutations (after copy number altering event) are identified as those occurring in diploid regions or regions of copy number amplification, but the 95% CI of the allele frequencies overlap 1 copy of the allele. Non-clonal mutations were defined as any mutation with an upper 95% CI allele frequency that falls below one copy indicating that it is present in a subpopulation of the cells. Proportion of each base substitution in each trinucleotide was calculated on a per patient per temporal category basis. Any patient with fewer than four somatic mutations in a particular category was excluded from the analysis to avoid potential biases.

### DNA constructs

Epitope (C-terminal 2xStrep-3xFlag)-tagged A3H-I and A3H-II constructs for purification from human cells were cloned as PCR fragments into pcDNA4/TO restriction sites HindIII-EcoRV using 5′-NNN-AAG-CTT-ATG-GCT-CTG-TTA-ACA-GCC-3′ and 5′-NNN-GAT-ATC-GGC-GGG-ACT-GCT-TTA-TCC-3′ and vectors described below as amplification templates. Catalytic mutant derivatives were constructed by standard site-directed mutation. The GST-A3H-I construct was made by PCR subcloning *A3H-I* cDNA from Open Biosystems (GenBank BC069023) using 5′-CCC GGG AAT TGG A AT GGC TCT GTT AAC-3′ and 5′-GCG GCC GC T CAG GAC TTT ATC CTC TC-3′ into SmaI/NotI digested pFastBac1-GST (Life Technologies). The A3B-HA, A3G-HA, A3H-I (untagged), and A3H-II (untagged) expression constructs are based on pcDNA3.1+ (Invitrogen). The A3B-HA, A3G-HA and untagged A3H-II were constructed by PCR subcloning coding sequencing matching Genbank accessions NM004900, NM021822 and FJ376614.1, respectively^71^,^75^. The untagged A3H-I construct was made by PCR subcloning *A3H-I* cDNA (GenBank FJ376611) using 5′-NNN-NGA-GCT-CGG-TAC-CAC-CAT-GGC-TCT-GTT-AAC-AGC-CGA-AAC-3′ and 5′-NNN-NGT-CGA-CTC-AGG-ACT-GCT-TTA-TCC-TCT-CAA-GCC-GTC-3′ into KpnI/XhoI-digested pcDNA3.1+ (Invitrogen). Sanger sequencing was used to confirm the integrity of all constructs.

### Protein purification and DNA deaminase activity assays

HEK293T cells were transfected with pcDNA4/TO-A3H-I:2xStrep3xFlag or pcDNA4/TO-A3H-II:2xStrep3xFlag (or catalytic mutants). Cells were harvested 48 h post-transfection and lysed in 50 mM Tris-HCl pH 8, 1% (v/v) NP-40, 150 mM NaCl, 0.5% (w/v) deoxycholate, 0.1% (w/v) SDS, 5 mM EDTA, 1 × EDTA-free Protease Inhibitor Cocktail (Roche), and then further disrupted by sonication. A3H proteins were purified using Strep-tactin resin (IBA). Samples were washed in high salt buffer (20 mM Tris-HCl pH 7.5, 1.5 mM MgCl_2_, 1 M NaCl, 0.5 mM DTT and 5% glycerol) followed by low-salt buffer (high salt with 150 mM NaCl) and elution using 2.5 mM desthiobiotin. Normalized amounts of each purified protein were incubated with a ssDNA substrate containing a target TCA motif and uracil DNA glycosylase for 1 h, and then treated with mild hydroxide to cleave the deaminated/de-uracilated substrate at the position of the abasic site as described^7^,^27^,^32^,^55^,^56^. The products of the assay were run on a 15% urea gel and were imaged using the Typhoon FLA 7000 fluorescent imager (GE Healthcare Life Sciences).

GST-A3H-I was produced using the Bac-to-Bac expression system (Life Technologies). *Sf9* cells were infected with recombinant GST-A3H-I baculovirus at an MOI of 20. Cells were harvested 40 h post-infection and lysed as described^76^. Cleared lysates were incubated with glutathione sepharose resin (GE Healthcare) and subjected to washes of PBS with 250 mM NaCl in the presence or absence of 1% Triton-X 100. The resin was then resuspended to a 50% slurry in 100 mM Tris pH 7.5, 100 mM NaCl, 10% glycerol, 1 μM ZnCl_2_ and 5 mM DTT. Aliquots of the 50% slurry with bound GST-A3H were incubated 2 h with 100 nM ssDNA substrate 5′-ATT-ATT-ATT-ANT-CAA-ATG-GAT-TTA-TTT-ATT-TAT-TTA-TTT-ATT-T-3′-fluorescein (N=A, C, G or T). Deamination events were detected by uracil DNA glycosylase and mild alkaline treatment as described^77^. Gel images were obtained using a FX fluorescence scanner (Bio-Rad) and background subtraction and integrated gel band intensities were quantified using ImageQuant (GE Healthcare). A3H-I showed no activity after elution of the enzyme from the affinity resin, and activity of the resin-bound enzyme diminished with time at 4 °C.

Virus infectivity and hypermutation experiments were done using 50% confluent 293T cells in DMEM (HyClone) with 10% FBS, and 0.5% Pen/Strep. Cells were co-transfected with 300 ng pΔNRF (gag-pol-rev-tat), 100 ng pMDG (vesicular stomatitis virus G protein), 100 ng pCS-CG (lentiviral transfer vector encoding green fluorescent protein (GFP)) and a titration (50, 100, 200 and 400 ng) of APOBEC3 expression plasmids (vector, A3G-HA, A3H-I or A3H-II) using TransIT-LT1 (Mirus Bio). After 24 h, the media was removed and replaced with fresh media containing 50 U ml^−1^ DNase to digest free plasmid DNA. After another 24 h, virus containing supernatants were purified with 0.45 μm polyvinylidene difluoride filters, treated with DNase at 50 U ml^−1^ and used to infect 293T target cells. Virus particles were isolated through a 20% sucrose cushion from the remaining supernatant. Producer cells were used for flow cytometry (FACSCanto II Ruo; BD Biosciences) and analysed (FlowJo) to monitor transfection efficiency. The remaining virus particles and producer cells were lysed in 2.5 × Laemmli sample buffer and used for immunoblotting. A3H was detected using a rabbit anti-A3H polyclonal antibody (Novus Biologicals, NBP1-91682), and A3A-HA and A3G-HA were detected using mouse anti-HA monoclonal antibody (Covance). Tubulin and p24 served as loading controls and were detected by a mouse anti-tubulin mononclonal antibody (Covance) and a mouse anti-p24 monoclonal antibody (NIH AIDS Reagent Program). Primary antibodies were detected with anti-rabbit and anti-mouse HRP-conjugated secondary antibodies (Jackson ImmunoResearch). After 48 h infection, target cells were harvested and used to analyse infectivity by GFP flow cytometry and to obtain genomic DNA for viral hypermutation analyses.

Genomic DNA from infected 293T target cells was used for viral hypermutation studies. Genomic DNA was first treated with DpnI (NEB) to remove plasmids that may have carried over from the original transfection. Phusion high-fidelity DNA polymerase (NEB) was used in nested PCR reactions to amplify a region in *GFP* with outer primers 5′-CCTRAARTTCATCTRCACCA-3′ and 5′-CACRCTRCCRTCCTC-3′, followed by the inner primers 5′-CCRCTACCCCRACCAC-3′ and 5′-TCACCTTRATRCCRTTCTTC-3′. Amplicons were cloned into pJET1.2 (Fermentas) and subjected to Sanger sequencing. Each sequence was aligned to the *GFP* reference sequence using BWA (ref. 78) and mutations were quantified using a custom perl script available from https://github.com/gjstarrett/countSangerMuts (Supplementary Software 1).

### Immunoblotting

Transfected 293T cells were lysed with Laemmli sample buffer and quantified for total protein by Lowry Assay (Sigma). Whole-cell lysate (35 μg) was separated on a 12% SDS–polyacrylamide gel electrophoresis. A3H was detected using a mouse anti-A3H mAb diluted 1:1,000 (ref. 48; NIH AIDS Reagent, Cat#12155). Alpha-tubulin was detected with a rabbit polyclonal serum (Thermo, PA1-20988). Fluorescent anti-mouse (680 nm, Licor, 926-68070) and anti-rabbit (800 nm, Licor, 32211) secondary antibodies were used for detection. Scans were performed on a Licor Odyssey infrared scanner.

### Immunofluorescent microscopy

Cell lines were obtained from the ATCC. HeLa cells were cultured in DMEM (HyClone) and SK-BR-3 and U2OS cells in McCoy's 5A (Corning), each supplemented with 10% foetal bovine serum and 0.5% Pen/Strep. 50% confluent cells were transfected with 200 ng of A3B-HA, A3G-HA, A3H-I or A3H-II with TransIT-LT1 according to the manufacturer's instructions (Mirus Bio). After 24 h incubation, cells were fixed in 4% paraformaldehyde and incubated overnight with either rabbit anti-HA (Cell Signaling, #3724; 1:250) or rabbit anti-A3H (Novus Biologicals, NBP1-91682; 1:200) followed by a 2 h incubation with anti-rabbit TRITC conjugated antibody (Jackson ImmunoResearch). The nuclei were stained using a 0.1% Hoechst solution. Cells were imaged using × 1,000 magnification on a Nikon Inverted TiE Deconvolution Microscope. Nuclear versus cytoplasmic localization of APOBEC3 proteins was calculated from these images using MATLAB (MathWorks) with the assistance of the University of Minnesota Imaging Center.

### Statistics

Statistical significance between categorical data was calculated by Welch's two-tailed *t*-test using both Graphpad Prism and the R statistical computing environment. Trinucleotide enrichments were calculated by Fisher's exact test.

### Data availability

Data that support this study were downloaded from https://confluence.broadinstitute.org/display/GDAC/Download, https://cghub.ucsc.edu or https://gdc.nci.nih.gov. All of the remaining data are included within the Article and its supplementary files or available from the author upon request.

## Additional information

**How to cite this article:** Starrett, G. J. *et al.* The DNA cytosine deaminase APOBEC3H haplotype I likely contributes to breast and lung cancer mutagenesis. *Nat. Commun.* **7,** 12918 doi: 10.1038/ncomms12918 (2016).

## Supplementary Material

Supplementary Information Supplementary Figures 1-7, Supplementary Tables 1.
