# Supplementary material for: The DNA cytosine deaminase APOBEC3H haplotype I likely contributes to breast and lung cancer mutagenesis
Source: Nat Commun. 2016 Sep 21;7:12918. doi: 10.1038/ncomms12918 (PMC5036005; doi:10.1038/ncomms12918)
Supplement: Supplementary Information — Supplementary Figures 1-7, Supplementary Tables 1. [file ncomms12918-s1.pdf]

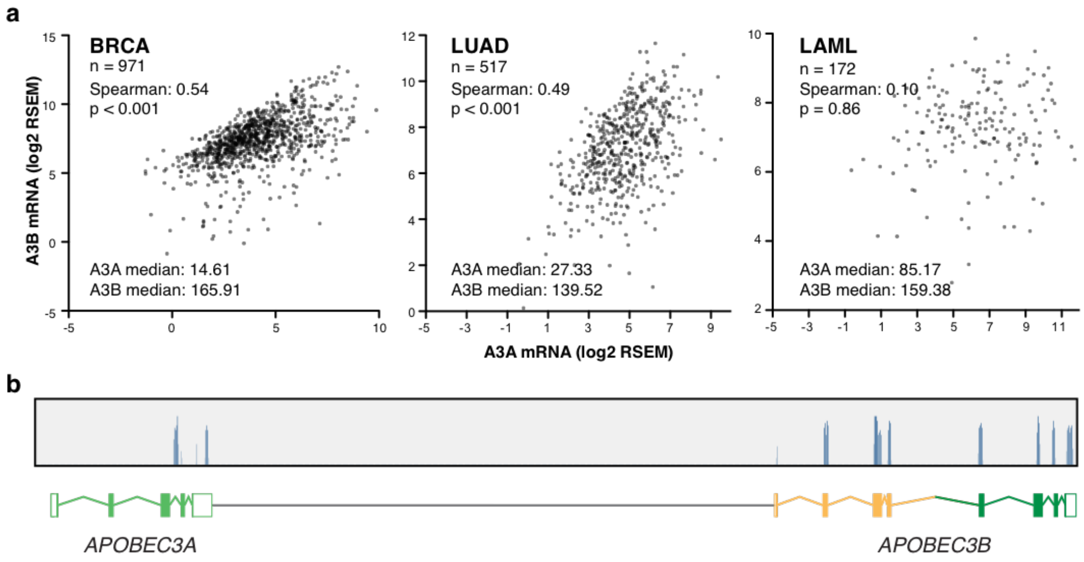

Supplementary Fig. 1. Evidence for cross-mapping of RNAseq reads from *A3B* to *A3A* in tumor samples impacting interpretations of mRNA expression.

a, A significant positive correlation is evident between *A3B* and *A3A* mRNA expression by RNAseq from tumors expressing high levels of *A3B* and low levels of *A3A* (*e.g.*, BRCA, breast invasive ductal carcinoma; LUAD, lung adenocarcinoma). In contrast, consistent with physiological expression of *A3A* in myeloid lineage cell types, the positive correlation disappears in acute myeloid leukemia, where *A3A* appears to be expressed at higher levels (LAML, acute myeloid leukemia).

b, Alignment of synthetically generated Illumina-like reads from *A3B* mRNA sequence mapping to *A3B* (as expected) and mis-mapping to *A3A* (~25%).

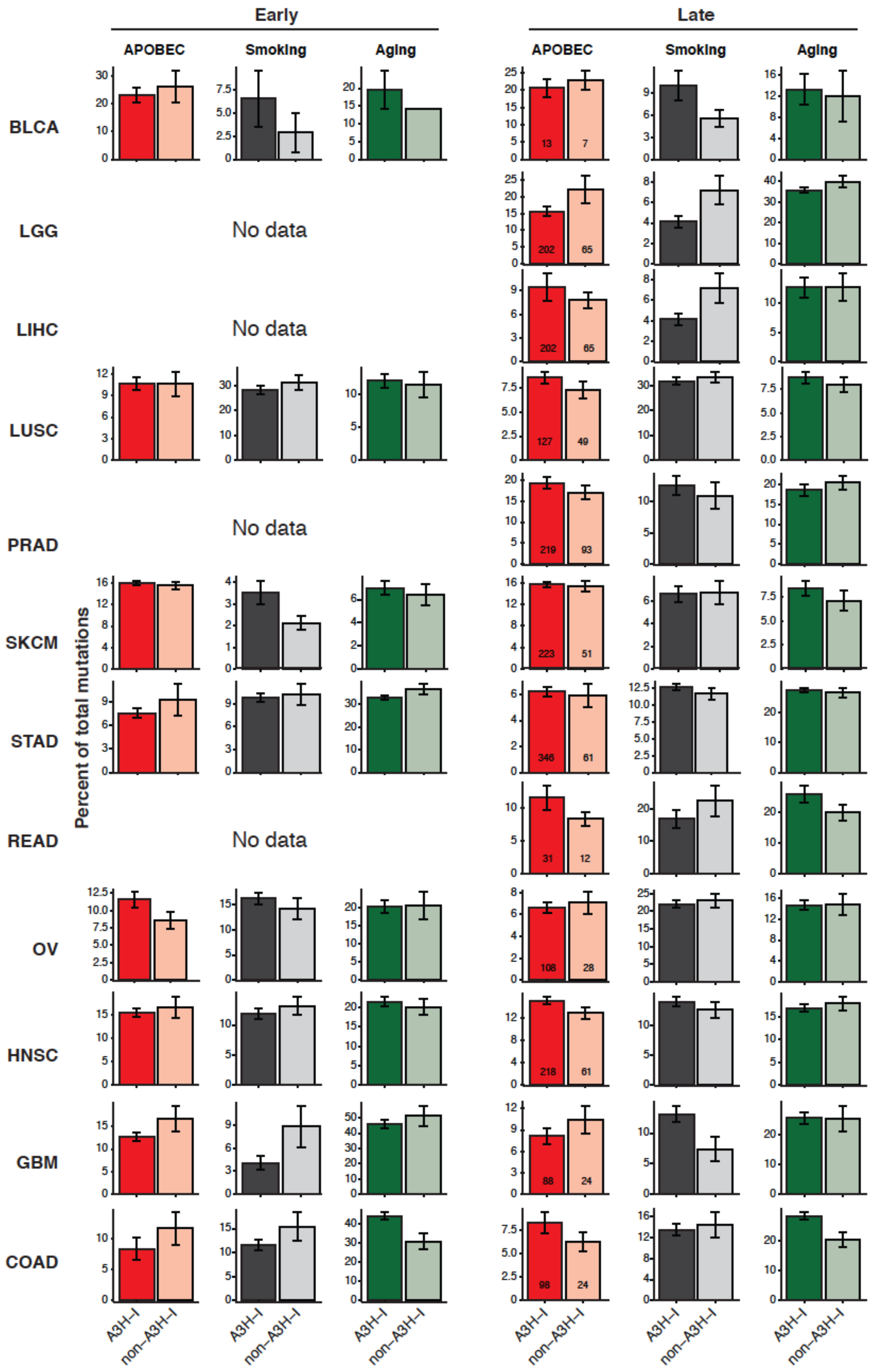
Early
Late
APOBEC
Smoking
Aging
BLCA
LGG
LIHC
LUSC
PRAD
SKCM
STAD
READ
OV
HNSC
GBM
COAD
No data
No data
No data
No data
Percent of total mutations
A3H-I
non-A3H-I

Supplementary Fig. 2. A3H-I and temporal separation of mutation signature associations in select TCGA cohorts.

Bar plots showing the frequency of clonal and subclonal mutations in the indicated cancer types attributable to APOBEC, smoking, and ageing (PRAD, prostate adenocarcinoma; BLCA, bladder urothelial carcinoma; SKCM, skin cutaneous melanoma; STAD, stomach adenocarcinoma; HNSC, head and neck squamous cell carcinoma; KIRC, kidney renal clear cell carcinoma; LGG, brain lower grade glioma; LUSC, lung squamous cell carcinoma; COAD, colon adenocarcinoma; READ, rectum adenocarcinoma; GBM, glioblastoma multiforme; THCA, thyroid carcinoma). Each bar represents the average proportion +/- SEM of signature mutations occurring within each *A3H* haplotype group (*i.e.*, *A3H-I* versus non-*A3H-I*). The total number of tumors with 1 or 2 copies of G105 (A3H-I) or 2 copies of R105 (A3H-II and other haplotypes) is indicated within the "Late APOBEC" set of histogram bars. Welch's two-tailed t-test for each category was used to calculate the p-values for all *A3H-I* and non-*A3H-I* pairings and none were significant for APOBEC mutation signatures. No data means either truly no available data in the indicated category or insufficient data for this analysis.

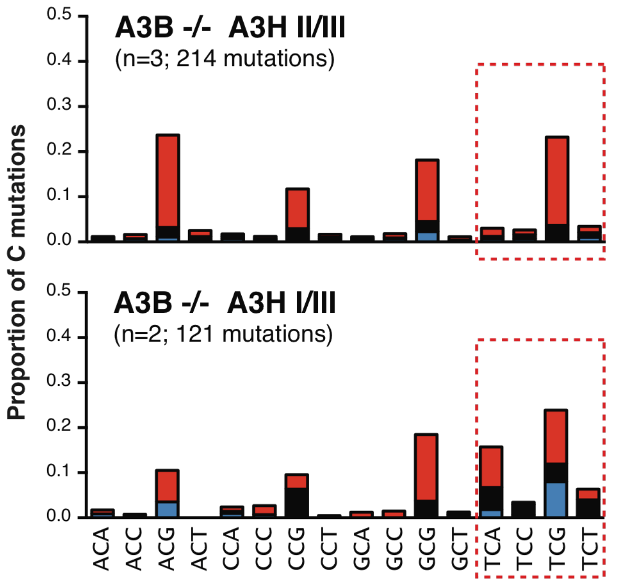

Supplementary Fig. 3. Weighted mutation distributions by *A3H* haplotype from TCGA breast cancer data (additional analysis of data presented in Fig. 1d).

Bar plots depicting the proportions of cytosine mutations occurring in the indicated trinucleotide motifs weighted by motif abundance in the human genome in *A3B*$^{-/-}$ breast cancers with the indicated *A3H* haplotype combinations (n-values and total mutation numbers in parentheses). C-to-T, C-to-G, and C-to-A are represented by red, black, and blue shading, respectively. Aging-related cytosine mutations in NCG motifs are prevalent in breast tumors regardless of *A3H* genotype, as expected. In contrast, APOBEC signature mutations are only visually evident in *A3B*-null tumors with *A3H-I* genotypes and, after correcting for motif abundance, TCG and TCA are mutated more than TCT, and TCC becomes the least mutated trinucleotide (concordant to biochemical data in Fig. 3e and HIV mutation data in Fig. 4c and weighted by motif abundance in Supplementary Fig. 4).

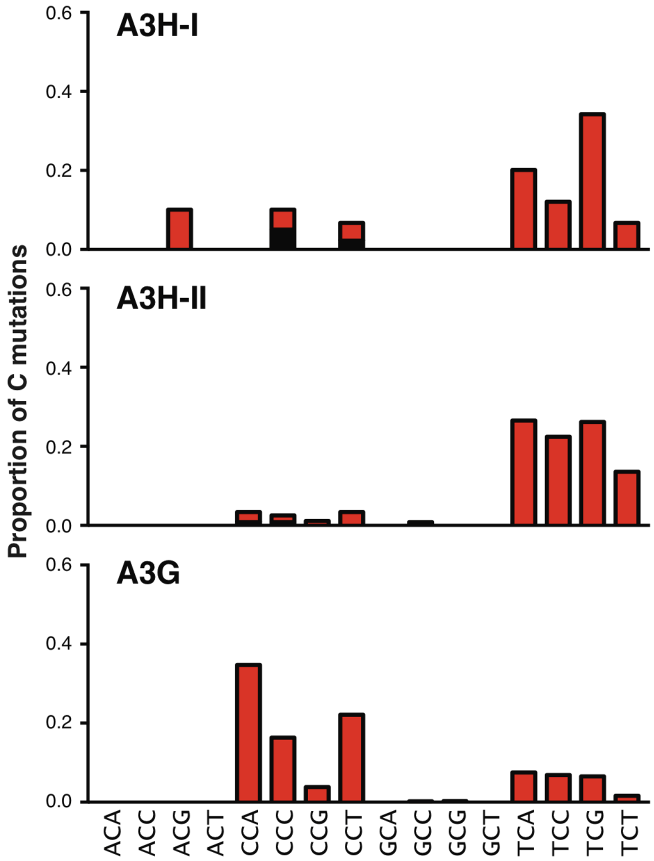

Supplementary Fig. 4. Weighted mutation distribution of mutations in viral DNA sequences (additional analysis of data presented in Fig. 4c).
C-to-T mutation distribution in viral DNA sequences weighted by motif abundance (NGN) in amplicon recovered from CEM-GFP reporter cells. C-to-T, C-to-G, and C-to-A are represented by red, black, and blue shading, respectively. The mutations are reported for the viral cDNA strand, rather than the conventional genomic strand to facilitate comparisons with tumor mutation data. After correcting for motif abundance, TCG and TCA are mutated more than TCT and TCC.

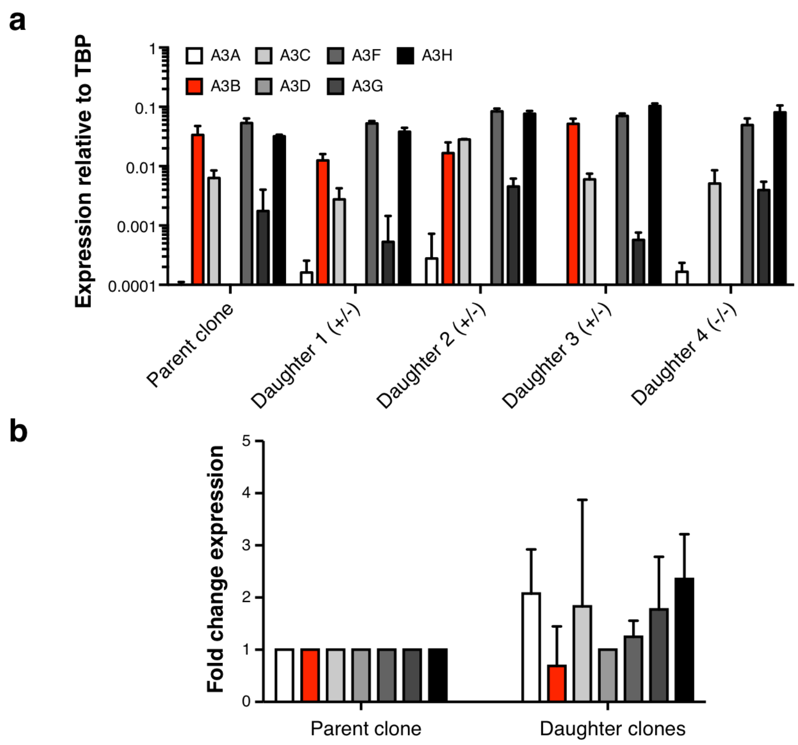

Supplementary Fig. 5. ***A3A*** **mRNA levels in MCF-7L cells and derivatives engineered by Cas9/CRISPR to have an** ***A3B*** **deletion identical to the naturally occurring deletion in humans.**

a, Expression of each *APOBEC3* family member relative to house-keeping gene, *TBP*, measured by RT-qPCR on a log10 axis for parent clone prior to engineering (*A3B* +/+) and 4 independent daughter clones after Cas9/CRISPR engineering (*A3B* +/- or -/-). Error bars show the standard error of 3 technical replicates.

b, Average fold change of *APOBEC* family member expression for daughter clones (right) targeted by Cas9/CRISPR relative to parent clone (left). Error bars show the standard error of the fold change in expression levels in the daughter clones (n=4; mean +/- SEM shown). The 1- to 2-fold differences seen here are not statistically significant and likely due to minor variations between clones.

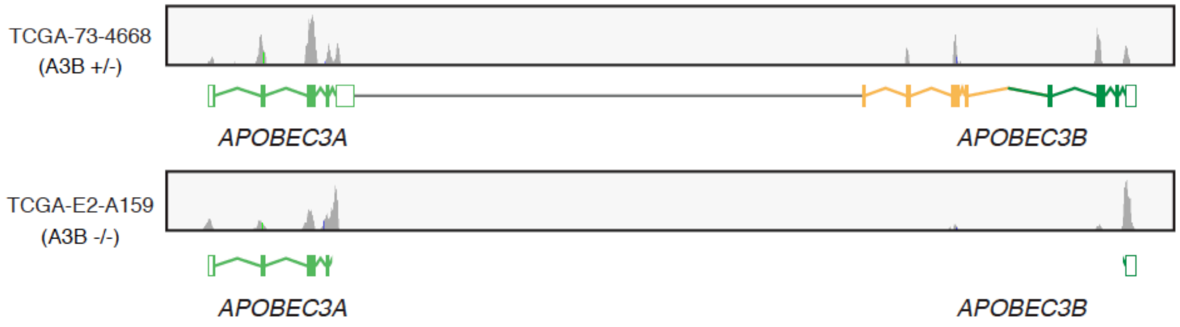

Supplementary Fig. 6. WES read coverage of *A3B* wild-type and *A3B*-null breast tumor specimens.
Representative depth of coverage histograms from WES alignments used to positively identify a representative *A3B* null patient. The top histogram shows peaks mapping to the *A3B* gene that, in the bottom histogram, are not seen in truly *A3B* null individuals.

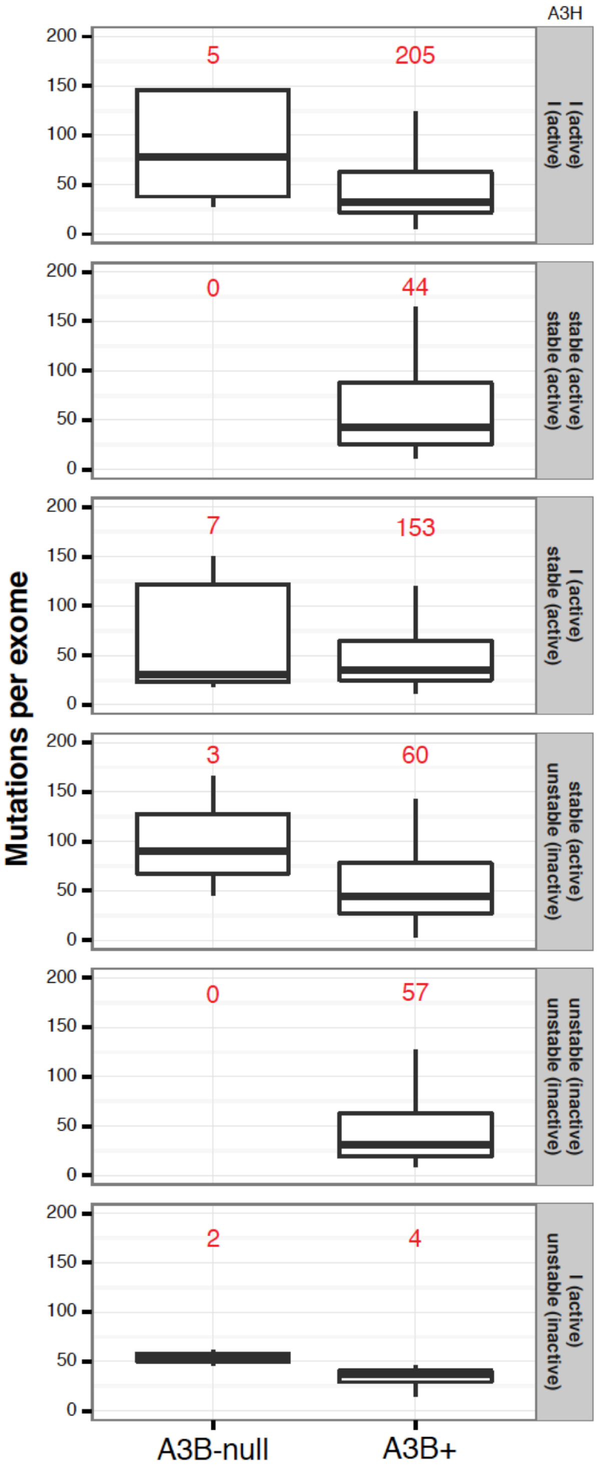

Supplementary Fig. 7. Exome mutation loads as a function of *A3B* and *A3H* genotypes. Whisker plots quantifying the number of mutations per tumor grouped by *A3B* and *A3H* genotypes. The total number of tumors with each genotype is represented by red numbers above each plots. The average is shown, the error box represents the first and third quartiles, and the whiskers extend to the highest value within 1.5x the interquartile range. Some genotypes are not represented in the entire TCGA breast cancer cohort.

Supplementary Table 1. Fisher's exact test for cytosine mutation types occurring in *A3H-I* tumors versus *A3H-II/III* tumors. This table is a statistical accompaniment to **Fig. 1d**. The proportion of each type of cytosine mutation in the A3H-II/III tumors was compared pairwise to the proportion of the same type of mutation in A3B-null breast tumors with the indicated A3H haplotypes.

Tumor A3H haplotype	C-to-T transition mutation motif				C-to-G transversion motif			
	TCA	TCC	TCG	TCT	TCA	TCC	TCG	TCT
**A3H-I/I**	8.50E-18	9.33E-06	1.08E-03	2.13E-07	1.14E-17	5.47E-05	3.18E-01	4.42E-17
**A3H-II/III**	1.80E-22	2.50E-05	3.39E-04	6.03E-10	5.87E-19	7.28E-03	2.11E-02	2.87E-15
**A3H-I/III**	5.83E-06	1.00E+0	1.00E+0	2.07E-01	3.68E-05	9.45E-02	1.00E+0	7.10E-03
